# A novel soluble epoxide hydrolase vaccine protects murine cardiac muscle against myocardial infarction

**DOI:** 10.1038/s41598-022-10641-x

**Published:** 2022-04-28

**Authors:** Takahiro Kitsuka, Aya Shiraki, Jun-ichi Oyama, Hironori Nakagami, Atsushi Tanaka, Koichi Node

**Affiliations:** 1 Department of Cardiovascular Medicine, Saga University, 5‑1‑1 Nabeshima, Saga, 849‑8501 Japan; 2 Department of Health Development and Medicine, Osaka University Graduate School of Medicine, Osaka, Japan

**Keywords:** Cardiovascular biology, Interventional cardiology, Cardiology

## Abstract

Myocardial infarction is still a life-threatening disease, even though its prognosis has been improved through the development of percutaneous coronary intervention and pharmacotherapy. In addition, heart failure due to remodeling after myocardial infarction requires lifelong management. The aim of this study was to develop a novel treatment suppressing the myocardial damage done by myocardial infarction. We focused on inhibition of soluble epoxide hydrolase to prolong the activation of epoxyeicosatrienoic acids, which have vasodilatory and anti-inflammatory properties. We successfully made a new vaccine to inactivate soluble epoxide hydrolase, and we have evaluated the effect of the vaccine in a rat myocardial infarction model. In the vaccinated group, the ischemic area was significantly reduced, and cardiac function was significantly preserved. Vaccine treatment clearly increased microvessels in the border area and suppressed fibrosis secondary to myocardial infarction. This soluble epoxide hydrolase vaccine is a novel treatment for improving cardiac function following myocardial infarction.

## Introduction

Cardiovascular disease (CVD) is still a major cause of mortality worldwide^1^, despite treatments such as percutaneous coronary intervention, preventive medicine, and best medical treatment in the chronic and post-myocardial infarction phase. In particular, percutaneous coronary intervention dramatically improved mortality from myocardial infarction^2^. However, the number of patients with heart failure (HF) associated with myocardial infarction (MI) has been increasing as the number of MI survivors increases. Therefore, it is important to minimize the extent of myocardial damage by MI to suppress myocardial remodeling and prevent the development of HF.

Epoxyeicosatrienoic acids (EETs) are cytochrome P450 2J2 epoxygenase metabolites of arachidonic acid^3^. These metabolites are known to potently promote anti-inflammation, vasodilation, fibrinolysis, anti-apoptosis, and angiogenesis^4^. We have been focused on EETs as promising candidates for cardioprotection since Koichi Node et al. reported their anti-inflammatory properties in addition to their vasodilatory action^5^. Gross et al.^6^ reported that EETs reduced ischemic size and area at risk in a myocardial ischemia model. Because soluble epoxide hydrolase (sEH) very quickly metabolizes EETs into dihydroxyepoxyeicosatrienoic acids (DHETs) that are not cardioprotective, several approaches have been made to enhance the activity of EETs by inhibiting sEH. Ex vivo studies in mice have demonstrated significant cardioprotective effects of not only EETs but also sEH inhibition against ischemia–reperfusion injury involving the PI3K pathway^7,8^.

Inhibition of sEH also exerted beneficial effects on cardiac function and post-MI ventricular remodeling, with direct positive effects on fibrosis and hypertrophy^7^. Furthermore, sEH inhibitors blocked NF-κB activation and reduced cardiac hypertrophy in the transverse aortic constriction (TAC) model of pressure-overload hypertrophy in mice^9^.

However, there are as yet no clinically approved sEH inhibitors for use in humans. Some candidates had poor metabolic stability, a relatively high melting point, and limited solubility in water, properties which made them difficult to use pharmacologically^10^.

Recently, the novel sEH inhibitor GSK22562940 proved to have a physiological effect, producing increased endothelial vasodilatory function^11^. However, its cardiac protective effect was comparatively modest, being less effective in reducing cardiac hypertrophy in the TAC model^12^.

Therefore, we decided to develop a vaccination system to inhibit the ability of sEH to reduce EETs activity. This study confirms that the sEH vaccine induced the production of antibodies in rats that inhibited sEH and increased the ratio of EETs to DHETs. The extent of myocardial infarction was correspondingly reduced in the vaccinated group, demonstrating the vaccine’s myocardial protective effect.

## Results

### Induction of antibody production by sEH vaccine

**Figure 1:**
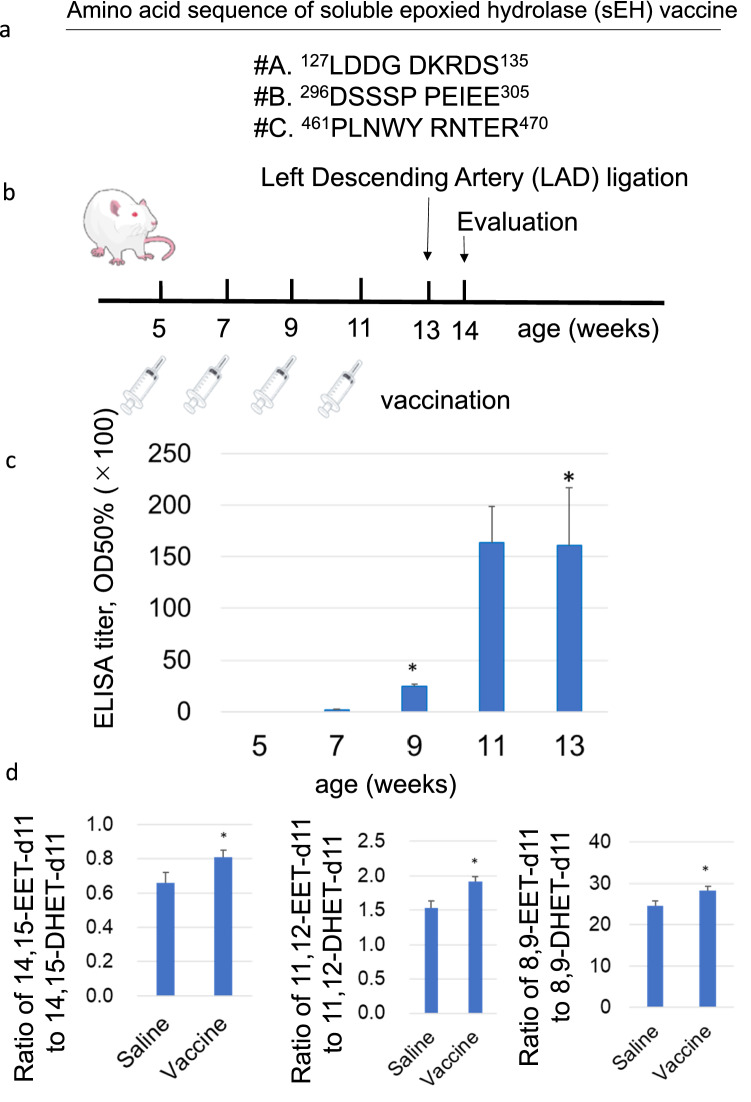
The antibody induced by sEH vaccine inhibited hydrolysis of EETs. (**a**) The peptide sequence of sEH vaccine. (**b**) The protocol of vaccination and evaluation. The sEH vaccine was administered to 5-wk-old rats 4 times at 2-wk intervals. (**c**) Anti-sEH antibody titer was quantified by ELISA. A high half-maximum value was obtained (> 1000) in all four rats after the second vaccination. n = 4. *P < 0.05. (**d**) Blood from rats was collected at 14 wk of age and preserved as plasma*. Having th*e antibody in the plasma prevented 14,15-EET-d11, 11,12-EET-d11 and 8,9-EET-d11 from being hydrolyzed into 14,15-DHET-d11, 11,12-DHET-d11 and 8,9-DHET-d11 and the ratio of 14,15-EET-d11 to 14,15-DHET-d11, 11,12-EET-d11 to 11,12-DHET-d11 and 8,9-EET-d11 to 8,9-DHET-d11 were increased in the vaccinated group. n = 8 in the control group and n = 9 in the vaccinated group. **P < 0.05.

Each peptide vaccine for rat sEH consisted of a short peptide sequence conjugated to keyhole limpet hemocyanin (KLH) as a carrier protein to induce the specific anti-sEH antibody. Knowing the sEH epitope information allowed three candidate peptides to be selected as antigens (#A, 127-135 LDDG DKRDS; #B, 296-305 DSSSP PEIEE; and #C, 461-470 PLNWY RNTER; Fig. 1a). The three peptide vaccines were conjugated to KLH and co-administered with Freund’s adjuvant t*o* 7-wk-old male C57BL/6NCrslc mice four times at two-week intervals (Supplementary Fig. 1a). Evaluation of antibody production by enzyme-linked immunosorbent assay (ELISA) showed that the titer against antigen peptide was successfully increased on days 42 and 56 in mice immunized with #A and on day 56 with #B vaccine; in contrast, no antibody for sEH was detected in mouse serum immunized by vaccine #C (Supplementary Fig. 1b). Therefore, we selected the #A and #B vaccines as candidates for further experimental studies. For this study, we chose the #A vaccine for its stronger titers.

The sEH peptide was conjugated with KLH and Freund’s adjuvant (complete at first vaccination, incomplete at second to fourth vaccinations). The sEH vaccine (6.25 μg/ rat) (n = 4) was administered to each rat at 5, 7, 9, and 11 weeks of age (Fig. 1b). To confirm the production of antibody in rats after sEH vaccination, antibody titers to sEH peptides were measured at 5, 7, 9, 11, and 13 weeks of age. Serum sEH antibody titers were not detected in any groups at five weeks of age when first vaccinated, but the sEH antibody titer increased at 9 weeks of age and was maintained until at least 13 weeks of age (Fig. 1c). Therefore, it was confirmed that antibody titer in rats treated with the sEH vaccine was well maintained during the experimental period.

### Confirmation of the antibody as neutralizing antibody

The degradation of 14,15-EET-d11 into 14,15-DHET-d11 was measured to determine the ability of the antibodies to inhibit sEH activity. We added 14,15-EET-d11 to hemolyzed whole blood of rats in control and vaccinated groups at 14 weeks of age and tested whether the elevated antibody could inhibit sEH activity. The amount of 14,15-EET-d11 and 14,15-DHET-d11 was detected and calculated using the LC–MS/MS system. This revealed that the ratio of 14,15-EET-d11 to 14,15-DHET-d11 was significantly higher in the sEH vaccine group and confirmed the inhibitory effect of antibody on sEH enzyme activity (Fig. 1d).

### sEH vaccine preserved cardiac function and suppressed cardiac remodeling after MI

**Figure 2:**
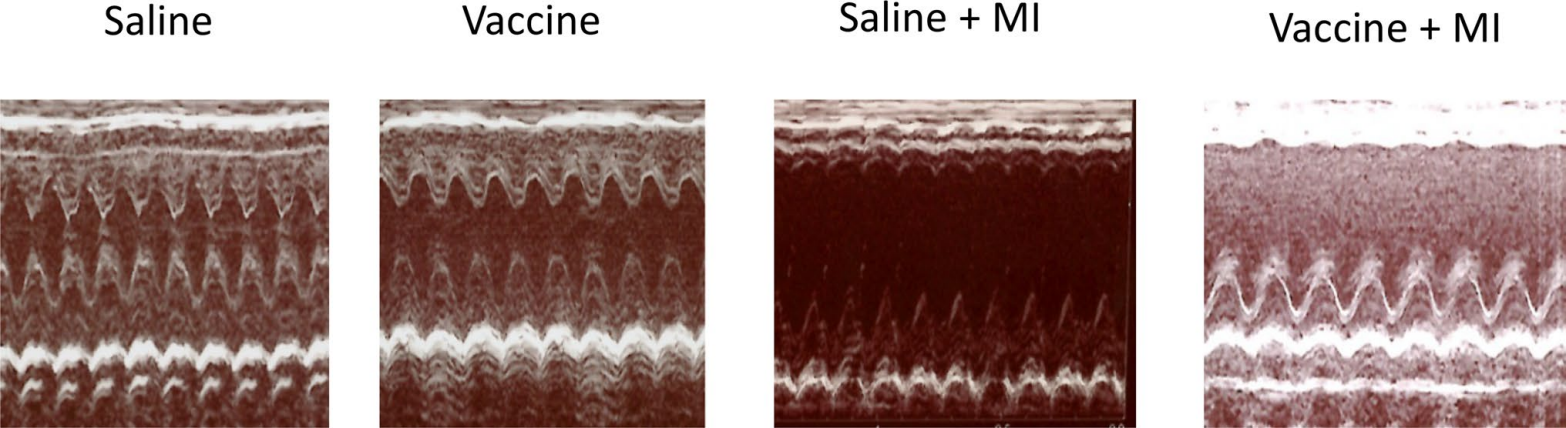
The sEH vaccine treatment improved cardiac function in MI model rats. Representative images of cardiac ultrasonography. Cardiac ultrasonography was carried out before MI induction (on the day of surgery) and after MI induction (7 days later).

We performed a total of 70 surgeries: saline (n = 40) or sEH vaccine (n = 30). Rats that died within 24 h were surgery-related deaths, and rats that did not show an EF reduction of 15% or more immediately after surgery were excluded from the study. Three rats died after 24 h, two in the saline group and one in the vaccine group. We analyzed a total of 51 rats that remained alive on day 7 after MI induction (n = 31 in saline, n = 20 in sEH vaccine). Transthoracic cardiac echocardiography was used to evaluate the effect of the sEH vaccine at baseline, 1 day and 7 days after MI induction (Fig. 2 and Table 1).

**Table 1:** Cardiac ultrasonography was carried out before MI induction (on the day of surgery) and after MI induction (1 day and 7 days later).

	Baseline				Post-day1				Post-day7			
	Saline (n = 5)		Vaccine (n = 7)		Saline (n = 4)		Vaccine(n = 5)		Saline (n = 8)		Vaccine (n = 13)	
LVAW (mm)	2.05	± 0.05	2.05	± 0.05	1.51	± 0.11	1.51	± 0.14	0.97	± 0.10	1.27	± 0.10*
LVDd (mm)	5.75	± 0.05	5.75	± 0.05	6.58	± 0.12	7.21	± 0.18*	7.83	± 0.23	7.14	± 0.31
LVDs (mm)	2.55	± 0.14	2.55	± 0.14	5.24	± 0.11	5.26	± 0.30	6.06	± 0.20	5.00	± 0.37*
EF (%)	89.8	± 1.3	89.7	± 1.4	47.4	± 3.4	57.8	± 4.6	49.5	± 3.2	62.0	± 3.9*
FS (%)	55.5	± 2.2	55.5	± 2.2	20.9	± 1.8	27.1	± 2.8	22.1	± 1.8	30.9	± 3.1*

Data shows no difference between the saline and vaccine groups at baseline and on post-day1. However, vaccination prevents the deterioration of cardiac function and myocardial remodeling on post-day7.

*MI* myocardial infarction, *LVAW* left ventricular anterior wall, *LVDd* diastolic dimension of left ventricle, *LVDs* systolic dimension of left ventricle, *EF* ratio of left ventricular ejection fraction, *FS* ratio of left ventricular fractional shortening.

*P < 0.05. Values were compared between the saline group and the vaccine group by Student’s *t*-test.

There was no significant difference at baseline before MI induction in systolic diameter of the left ventricle between the preoperative saline group and the vaccine group. The ejection fraction (EF) and fractional shortening (FS) were also not different at baseline and day1 after MI induction (Table 1). The fact that there were no difference baseline values between the saline group and the vaccine group indicated that vaccination itself did not affect cardiac function in rats. On day1, LAD ligation caused deterioration of cardiac function in both groups.

At 7 days after surgery, the systolic dimension of the left ventricle (LVDs) in the vaccinated group was smaller than in the saline group. The left ventricular anterior wall (LVAW) in the saline group was thinner than that in the vaccinated group, indicating that the sEH vaccine significantly preserved LVAW after MI (P < 0.05). Both FS and EF were increased in the vaccine group compared to the saline group (P < 0.05).

Taken together, the sEH vaccine suppressed the dilation of the left ventricle due to myocardial infarction and improved systolic function.

### sEH vaccine reduced the fibrosis and infarct size in cardiac muscle after MI

**Figure 3:**
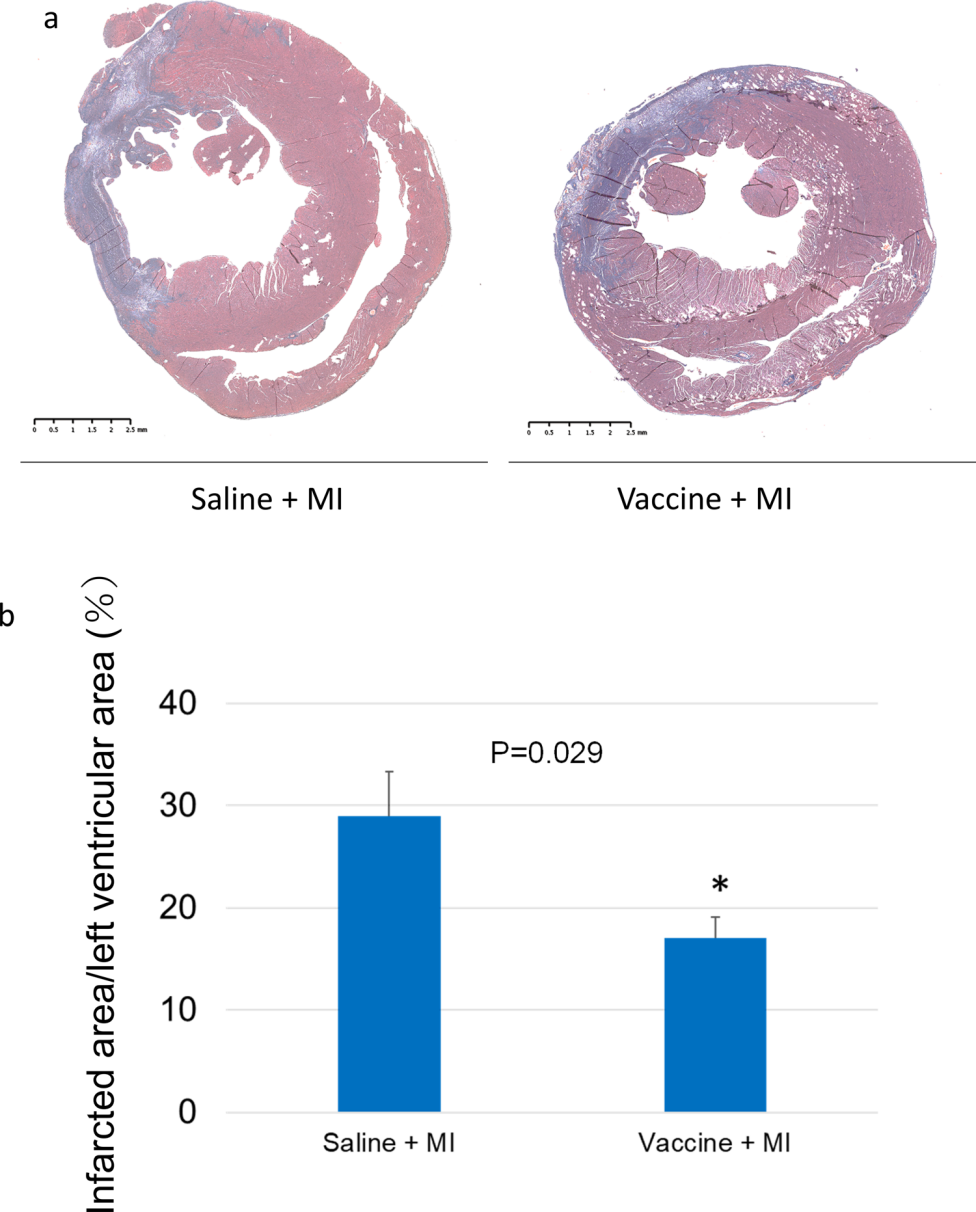
The sEH vaccine treatment reduced infarct size and fibrosis in cardiac tissue. (**a**) Cardiac sections were evaluated by Azan staining to determine the amount of fibrosis induced by MI after one week. The blue area indicates fibrosis due to myocardial infarction, and the red part indicates the non-infarcted area. (**b**) Infarcted area ratio was calculated by the fibrotic area / whole left ventricular area that shows the ratio of the infarcted tissue in the short axis sections. The vaccinated group had significantly reduced infarcted area. Saline group (n = 4), vaccinated group (n = 5), *P < 0.05. Percent myocardium infarcted was calculated in the saline and vaccinated groups.

Azan staining in the short axis of cardiac muscle showed that infarct-induced fibrosis was significantly lower in the vaccinated group than in the saline group (16.6% vs. 28.8%, respectively; P = 0.029, Fig. 3). These results indicate that the anti-sEH vaccine suppressed the amount of myocardial damage associated with myocardial infarction.

### sEH vaccine increased capillary density and the number of arteries after myocardial infarction

**Figure 4:**
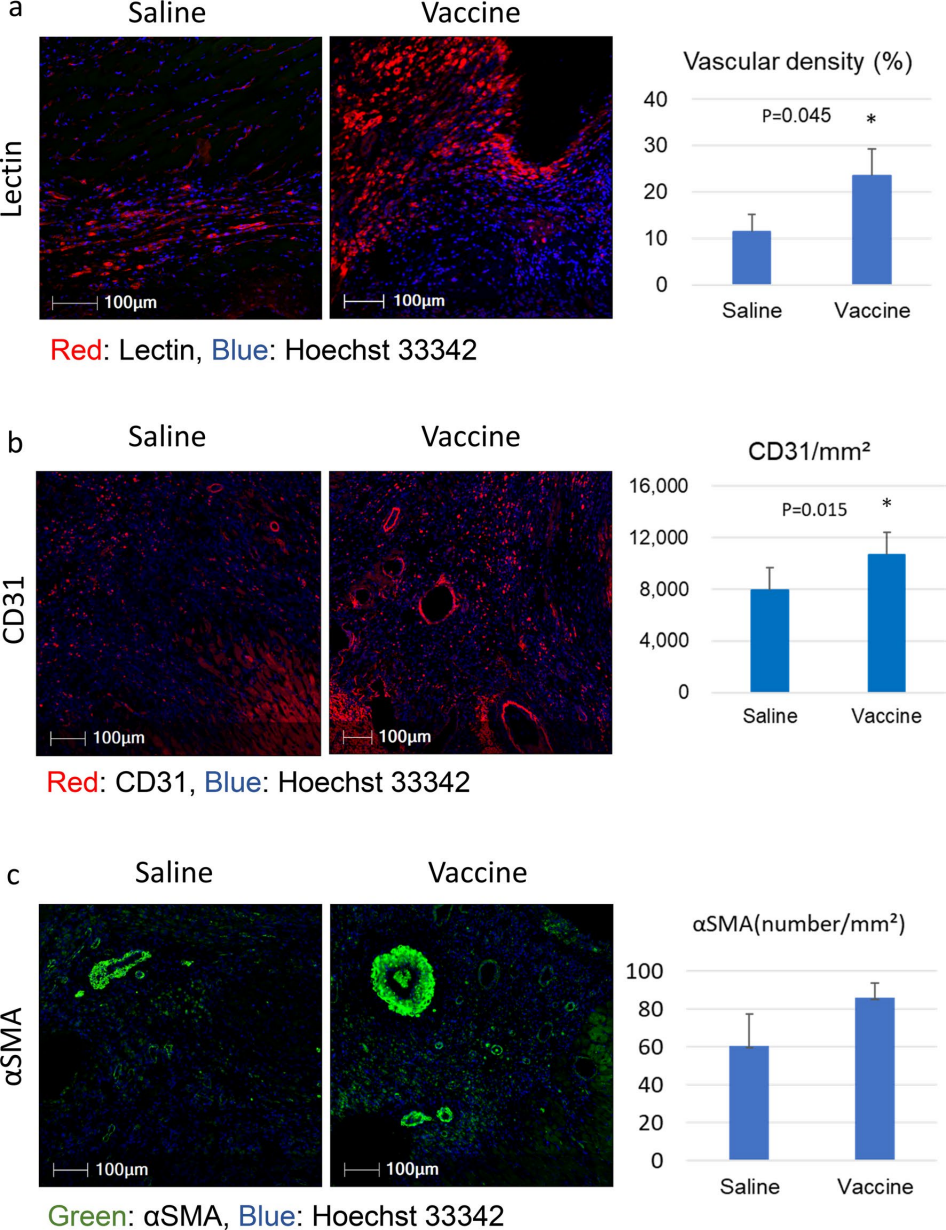
sEH vaccine treatment induced neovascularization in ischemic border area. (**a**) Immunofluorescent staining of (**a**) lectin, (**b**) CD31, and (**c**) α-smooth muscle actin. The nuclei were visualized using Hoechst 33342. Sections of hearts from the saline group (n = 5–7) and the vaccinated group (n = 5) at day 7 were evaluated. Scale bar = 100 μm. The border areas between infarcted and non-infarcted sites were randomly measured at five locations. Cells labeled with CD31, a marker of endothelial cells, were counted (cells/mm^3^). *P < 0.05 Lectin was also used to assess vascular density (%). *P < 0.05.

Intramyocardial arteries and capillaries in the border zone on 7 days after MI were assessed. For labeling, we used lectin specialized to bind specific oligosaccharide side chains of vascular endothelial cells (Fig. 4a), anti-CD31 to label endothelial cells (Fig. 4b), and anti-α-smooth muscle actin to label smooth muscle cells (Fig. 4c). Five areas (0.1 mm^2^ each) in the border area were randomly chosen and the labeling densities were measured.

The number of myocardial vessels including capillaries and arteries in the sEH vaccine group was significantly greater than that in the saline group, suggesting that sEH inhibition promoted angiogenesis in the border area between the ischemic and non-ischemic areas.

### sEH vaccine induced vascular endothelial growth factor (VEGF) and endothelial nitric oxide synthase (eNOS)

**Figure 5:**
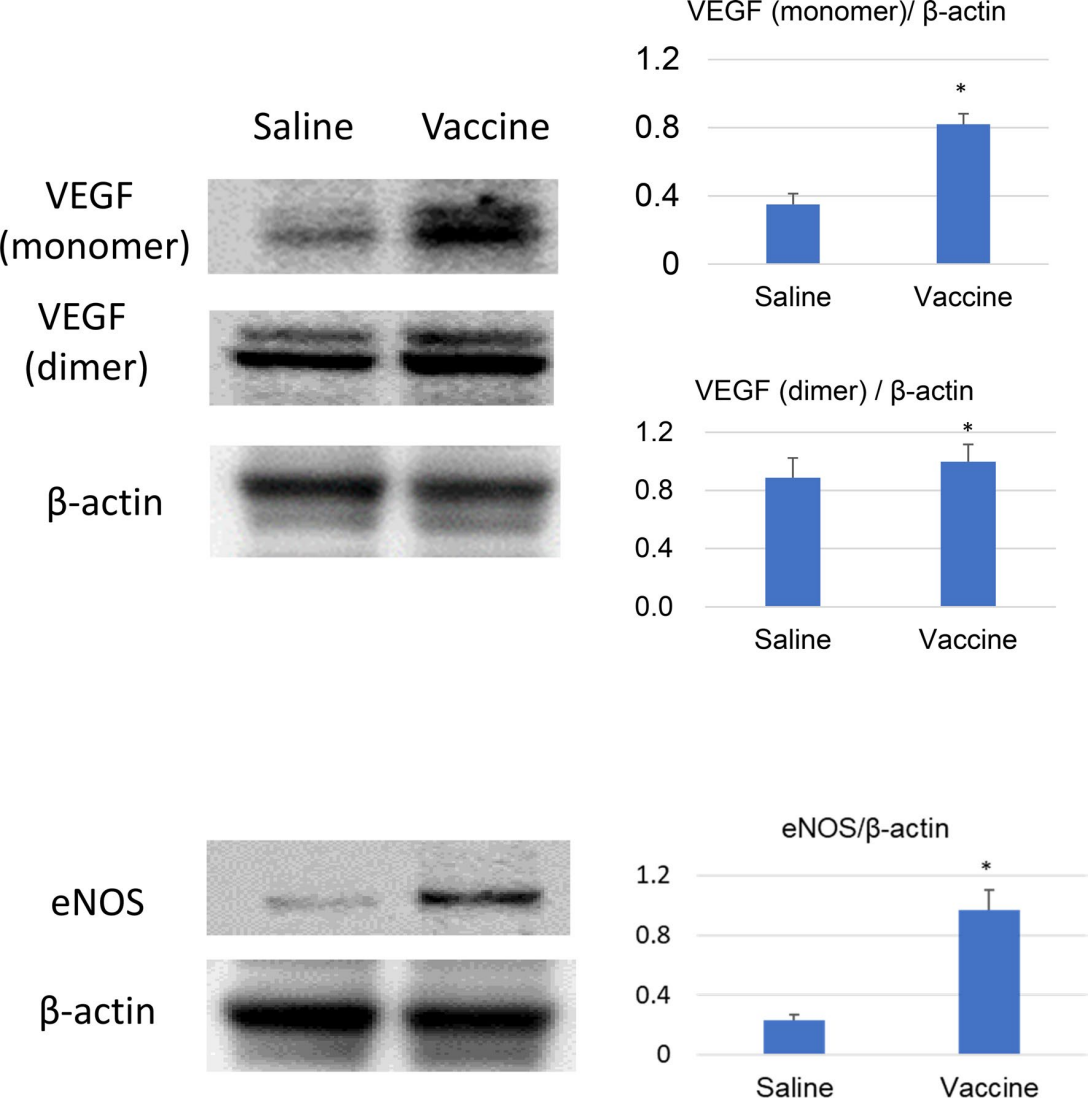
sEH vaccine treatment upregulates VEGF and eNOS. Vascular endothelial growth factor (VEGF) and endothelial nitric oxide synthase (eNOS) in the heart were analyzed by Western blot. β-actin was used as a control. Saline group (n = 4), vaccine group (n = 4). *P < 0.05.

We hypothesize that VEGF was the main cause of angiogenesis because EETs are known to promote angiogenesis through VEGF^13,14^. In confirmation, the protein expression level of VEGF in the vaccinated group was increased compared to that in the saline group (P = 0.040, Fig. 5).

In addition, the levels of eNOS were also significantly upregulated in the sEH group (P = 0.004, Fig. 5).

### Examination of vaccine safety

**Figure 6:**
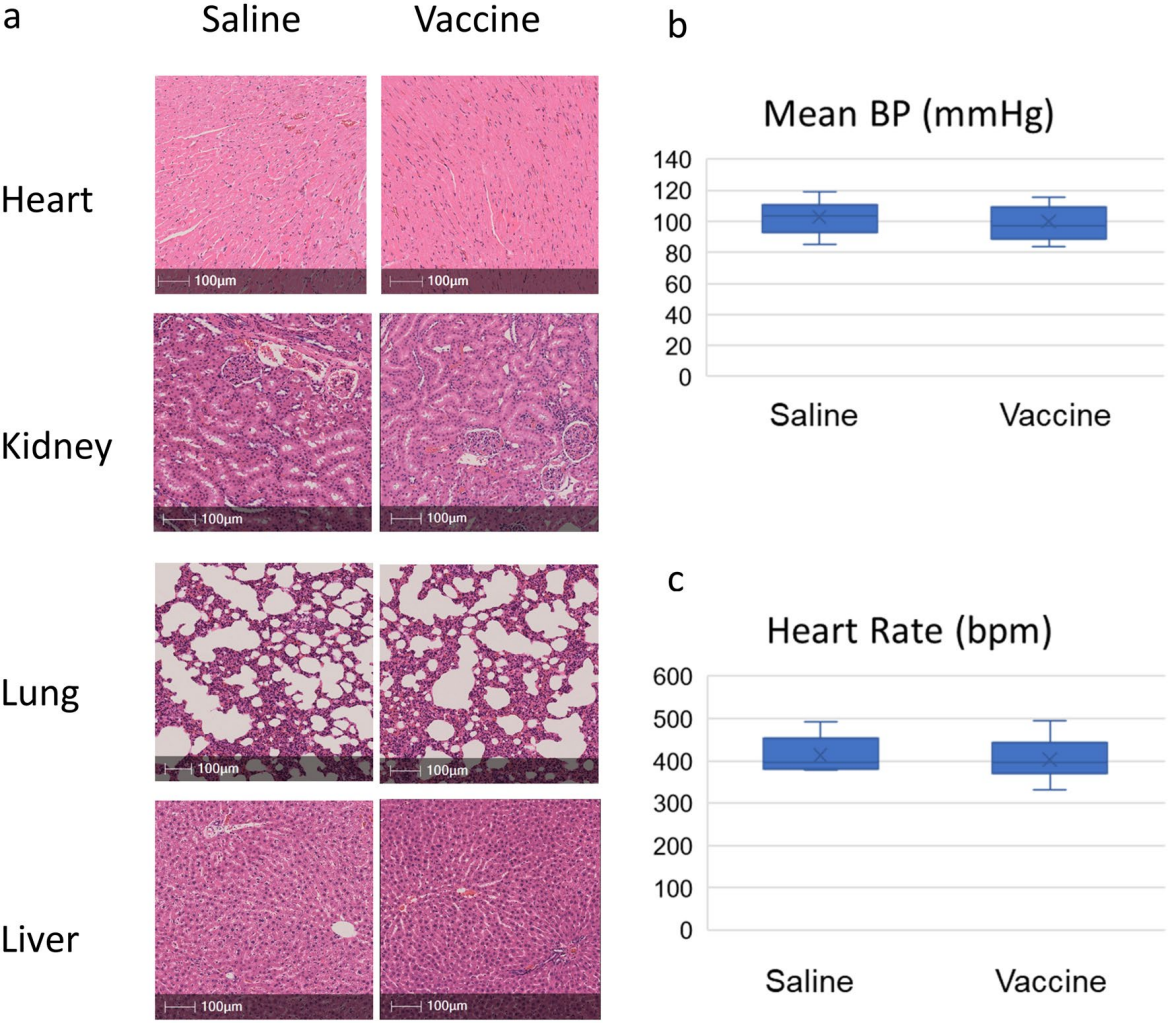
sEH vaccine was safely administrated in rats. (**a**) A representative photomicrograph of hematoxylin and eosin staining in heart, lung, kidney, and liver. Scale bars = 100 μm. (**b**) The mean blood pressure at 11 weeks of age before MI induction. Saline group (n = 10), vaccine group (n = 11). (**c**) The mean heart rates at 11 weeks of age before MI induction. Saline group (n = 10), vaccine group (n = 11).

As shown in Fig. 6a, treatment with the sEH vaccine induced no visible pathological changes in heart, lung, kidney, or liver at 13 weeks of age.

We also evaluated the effect of vaccination on blood pressure at 11 weeks of age; no significant difference was detected in blood pressure between the control group and the vaccinated group (102.8 ± 3.2 vs 99.5 ± 3.6 mmHg; P = 0.51, Fig. 6b). There was also no significant difference in heart rate between the two groups (413.2 ± 12.8 beats/min vs. 403.0 ± 13.6 beats/min; P = 0.59).

From the results above, sEH vaccine administration was safe and did not affect hemodynamics (Fig. 6c).

## Discussion

In this study, we have shown that the sEH vaccine was safe and induced neutralizing antibody in rats. The antibody inhibited sEH and increased the EET/DHET ratio. Furthermore, the sEH vaccine reduced the size of the ischemic zone in MI induced by LAD ligation in rats. The reasons for myocardial protective effects may include VEGF and eNOS upregulation. The rise of EETs has already been reported for angiogenetic and myocardial protective effects occurring together with VEGF and eNOS elevation^13–16^, and our data are consistent with those studies.

Both of eNOS and VEGF are angiogenetic factors. The transfection of eNOS DNA into a rat ischemic hindlimb led to significant increase in blood flow and capillary number accompanied with VEGF up-regulation^17^. It is known that VEGF-Akt signaling also activate eNOS expression and induce vasodilation through NO production^18^. We speculated that the increase in capillaries was obtained by upregulation of VEGF and eNOS through EETs activated by sEH inhibition.

There are some reports that influenza vaccines have reduced the mortality rate due to myocardial infarction^19–21^. In contrast, we newly developed a vaccine specific to enzymes in the myocardium in this report.

**Figure 7:**
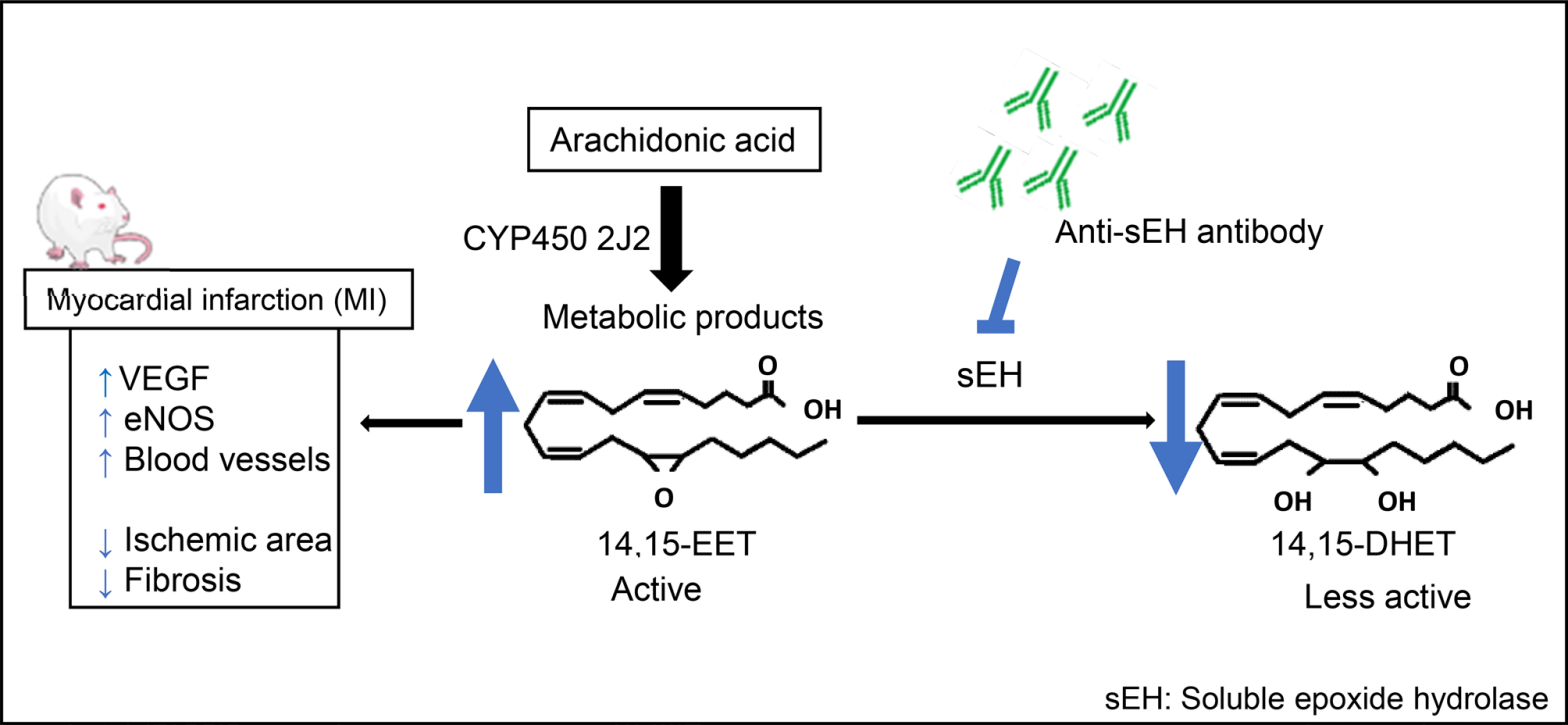
Schematic figures depicting the mechanisms of how sEH vaccine works in the MI model. *sEH* Soluble epoxide hydrolase, *CYP450* Cytochrome P450, *EET* epoxyeicosatrienoic acid, *DHET* dihydroxyeicosatrienoic acid, MI: myocardial infarction, *VEGF* vascular endothelial growth factor, *eNOS* endothelial nitric oxide synthase.

This study shows that this vaccine therapy improves cardiac function and suppresses adverse pathological changes in the rat MI model (Fig. 7). In general, permanent ligation leads to severe cardiac ischemia and there are some acute deaths, however, the mice who survived through acute phase can be evaluated for myocardial infarction^22–25^. LAD permanent ligation results in most of the area at risk being infarcted, leading to significant cell death and a large scar^26^. The Present sEH vaccine improved such severe ischemic model, suggesting its potent cardioprotective effects through neovascularization.

The idea of vaccine therapy for non-infectious diseases is novel, and there are only a few researchers who have adopted this approach. Previous studies showed that a similar peptide-KLH conjugate, the Ang II vaccine, induces the production of anti-Ang II antibodies and has therapeutic effects on animal models of hypertension^27^ and myocardial infarction^28^. Also, Yoshida et al.^29^ developed an anti-CD153 vaccine targeting senescent T cells as a senotherapeutic option.

Several reports have shown beneficial effects of increasing EETs in mouse models of LAD ligation. The perfused hearts from sEH-null mice showed greater recovery from LAD ligation for 20 min than those from wild-type mice through PI3K-GSK-3β signaling and K_ATP_ channels^8^. The isolated hearts from transgenic mice with cardiomyocyte-specific overexpression of CYP2J2, which upregulates EETs, also showed cardioprotective effects against ischemic-reperfusion injury through PKA activation and K^+^ channels^30^. Two kinds of sEH inhibitors, 1-adamantan-1-yl-3-{5-[2-(2-ethoxy-ethoxy)-ethoxy]-pentyl}-urea (AEPU) and trans-4-[4-(3-adamantan-1-yl-ureido)-cyclohexyloxy]-benzoic acid (t-AUCB), successfully reduced infarct size and inhibited the progression of cardiac remodeling in an ischemia–reperfusion model by suppressing inflammation^31^. Another sEH inhibitor, 12-(3-adamantane-1-yl-ureido)-dodecanoic acid butyl ester (AUDA-BE), also reduced ischemic injury in an ischemic-reperfusion model in mice^32^ even though it was administered intraperitoneally. Our results showing that the sEH vaccine attenuated myocardial infarction are consistent with those preceding reports.

The difference between these publications and our experiments is the accessibility of the sEH by the sEH inhibitory agents. Because sEH is known as an intracellular protein, it is thought to be difficult to for the neutralizing antibody access sEH protein directly though membrane of the lipid bilayer. We postulated that cell membrane could loose and sEH was leaked from the cardiac muscle when the cells went necrosis as well as creatine phosphokinase, troponin, lactate dehydrogenase, aspartate aminotransferase and alanine aminotransferase during process of MI. In fact, we found that sEH were diffusely stained in the cardiomyocytes (Supplementary Fig. 3a) but not in the infarcted area. In western blotting, the sEH protein was also remarkably decreased in the infarcted area (Supplementary Fig. 3b). The exact sEH concentration in the blood could not be measured because there is no system to detect low level of rat sEH. We, however, found the ratio of 14,15-EET-d11/14,15-DHET-d11 was significantly decreased compared to the baseline (Supplementary Fig. 4), that was analyzed using the blood in 4 h from LAD ligation. This result suggests that sEH probably be present and activated in the blood after myocardial infarction, making it more accessible to antibodies.

VEGF is a strong and essential proangiogenic factor that promotes angiogenesis after acute myocardial infarction^33^. Our previous study showed that the novel VEGF mimic COA-Cl reduced infarct area in an MI model mice^34^. Our sEH vaccine increased EETs and upregulated VEGF, which is compatible with reports of other researchers. For example, Zhan et al.^35^ reported that sEH inhibition augmented VEGF production after oxygen‐glucose deprivation in cultured astrocytes, and Suzuki et al.^36^ used a reporter gene assay to show that sEH inhibition promoted VEGF expression in human umbilical artery endothelial cells under hypoxia. VGEF is a known angiogenetic factor and plays a protective role against cardiac cell death induced by hypoxia^37^. We also proved that the sEH vaccine increased capillary and vessels in the border area. Based on these findings, VGEF induced by sEH inhibition is suggested to have rescued cardiomyocytes at the border of the ischemic area in our experiments.

Vaccination produced no adverse effects on cardiac function or histopathological changes in heart, lungs, liver, or kidneys. These results suggest that the sEH vaccine had no apparent organ-toxic effects, although it did induce lumps and hardness at the injection site in some rats. It is also reported that observation studies of sEH-null mice revealed only mild weight loss, with no reports on development of cancer^38^. However, some reports indicate that inhibiting sEH can lead to cancer metastasis^39^. For future clinical application, the vaccine may need to be used only after the patients have been carefully examined for cancer.

The late side effects of the vaccine will need to be validated over a longer observation period and it will be necessary to evaluate how long the antibody titer lasts. Furthermore, we analyzed only the rate of 14,15-EET to 14,15-DHET conversion, but sEH also hydrolyzes EETs such as 11,12-EET, 8,9-EET, and 5,6-EET. The sEH vaccine is expected to upregulate other EETs as well.

EETs are reported to cause hyperpolarization and relaxation of vascular smooth muscle. EETs act as an endothelium-derived hyperpolarizing factor (EDHF) in many vascular beds, including the coronary artery and renal circulation, and lowers blood pressure^40^. Inhibiting sEH has been reported to be effective in lowering blood pressure in a pathological hypertension model^41^. In our study, however, there was no significant reduction in blood pressure in the vaccinated group. Possible reasons for this discrepancy could be that the model we used was not a pathological hypertension model but rather a healthy sham or MI model.

For clinical application in humans, after first confirming that antibodies capable of inhibiting sEH can be raised in human blood, patients with overlapping risks of atherosclerosis, such as those who are prone to myocardial infarction due to diabetes, hypertension, and/or obesity, may be eligible for the sEH vaccine. In addition, since those who have had a previous myocardial infarction are prone to recurrence, sEH vaccination at the time of the first event may be cardioprotective. Vaccine effects are maintained far longer compared with oral dosing of conventional drugs, and the vaccine could be purified at a lower cost than long-term use of conventional drugs.

In conclusion, we have demonstrated that the sEH vaccine has a protective role in acute myocardial infarction, showing reduced ischemic area and fibrosis. The sEH vaccination upregulates VEGF and increases vascularity in cardiac muscle after MI in rats. We propose that the sEH vaccine is a novel candidate for a preventive treatment to reduce myocardial damage after MI.

## Materials and methods

### Vaccine design, peptide synthesis, and preparation

Based on high antigenicity analysis, we predicted qualified peptide structures from epitope information. We selected one of three different antigenic peptides from the amino acid sequence of mouse sEH (#A, 127-135 LDDG DKRDS; #B, 296-305 DSSSP PEIEE; #C, 461-470 PLNWY RNTER). After Cys was added to the N-terminus of each peptide, N-(6-maleimidocaproyloxy) succinimide was used to conjugate each peptide to KLH (Peptide Institute Inc., Osaka, Japan)^27^. We used these vaccines with complete Freund’s adjuvant for the first vaccination, and we additionally injected the vaccines with incomplete Freund’s adjuvant three times at two-week intervals (total 4 times). The sEH peptide vaccines consisted of 0.13 mg/mL sEH peptide and 0.87 mg/mL KLH in sterile PBS.

### Enzyme-linked immunosorbent assay (ELISA)

The serum sEH antibody titer was quantified by ELISA as previously reported^29^. The candidate antigenic peptide-BSA conjugate (Peptide Institute Inc., Osaka, Japan) was coated at a 10 μg/ml concentration and diluted in 50 mM carbonate buffer overnight at 4 °C on 96-well ELISA plates (MaxiSorp Nunc, Thermo Fisher Scientific K.K., Japan). After blocking with PBS containing 5% skim milk, the sera were serially diluted in blocking buffer, added to each well, and incubated overnight at 4 °C. After washing each well with 0.05% PBS Tween-20 (PBS-T), the cells were incubated with horseradish peroxidase-conjugated antibodies specific for mouse IgG (1:1000; GE Healthcare, UK) for mouse serum, or with rat IgG (1:1000; GE Healthcare, UK) for rat serum, for 3 h at room temperature. After washing the wells with PBS-T, color was developed with the peroxidase chromogenic substrate 3,3’,5,5’-tetramethylbenzidine (TMB; Sigma Aldrich, MO, USA), and the reactions were terminated with 0.5 N sulfuric acid. The absorbance was measured at 450 nm using an Immunomini NJ-2300 (BIOTEC, Japan). The half-maximal antibody titer was determined according to the highest value in the dilution range of each sample.

### Sample preparation for LC–MS/MS

Rat plasma including antibodies that were preserved at the day7 from LAD ligation was used. The hemocytes including rat sEH protein were collected by fresh blood from one alive rat. 30 μL of dH_2_O was added to an equal volume of hemocytes to hemolyze and 30 uL of plasma was also added. 14,15-EET-d11 (Item No.10006410, Cayman) 11,12-EET-d11 (Item No.10006413) and 8,9-EET-d11 (Item No. 10009998) at 2.2 ng/μL (final concentration), and vortexed. After 15 min incubation at 37 °C, 3 mM zinc sulfate was added to stop the reaction and samples were preserved at − 20 °C. Stocked samples were extracted three times with the same amount of ethyl acetate. The organic phase was collected and evaporated by miVac Duo (Genevac, UK).

### LC–MS/MS conditions

#### Chromatographic conditions

We used LCMS-8030 (Shimazu, Japan). A Sim-pack XR-ODSIII 2.2 μm C18 150 mm × 2.0 mm HPLC column (Shimazu, Japan) was used for HPLC separation. The column was kept at 40 °C. Mobile phase A was 0.1% formic acid in water, and mobile phase B was acetonitrile. LC gradient was as follows: 45%B → 75%B (15 min) → 75%B (15 min) → 45%B (15 min) → 45%B (10 min). The flow rate was 0.2 mL/min. A typical injection volume was 18 μL using partial loop injection mode.

#### Mass spectrometric conditions

We used LCMS-8030 with an electrospray ionization interface (ESI), operating in negative mode. The instrument was optimized by infusing a 0.1 μg/mL solution of 14,15-EET-d11 and 14,15-DHET-d11 (Item No.10008040, Cayman) in acetonitrile at 10 μL/min with a flow of 0.2 mL/min 25/75–0.1% formic acid in water/acetonitrile from the LC compartment into the mass spectrometer. The multiple-reaction-monitoring (MRM) transitions of m/z 330 → 219, and m/z 348 → 207 were chosen for 14,15-EET-d11 and 14,15-DHET-d11, respectively. We selected the MRM transitions from signal to noise ratio and selectivity. The MRM transitions for 14,15-DHET-d11 were acquired at 1.7 min. MRM transitions for 14,15-EET-d11 were acquired at 2.5 min. The DL temperature was 300 °C. Data were processed using Labsolution software (Shimazu, Japan). Calibration plots of 14,15-DHET-d11 and 14,15-EET-d11s were constructed. The ratio of 14,15-EET-d11 to 14,15-DHET-d11 in each sample was calculated.

### In vivo experimental protocols

All experiments followed the ARRIVE guidelines and were performed in accordance with the “Position of the American Heart Association on Research Animal Use” and approved by the Institutional Animal Care and Use Committee at Saga University. Wistar rats were purchased from Japan SLC. Rats were randomly assigned to two groups: a saline group (n = 40) and a vaccinated group (n = 30) for MI evaluation, and a saline group (n = 4) and a vaccinated group (n = 6) for vaccine safety evaluation. Rats were vaccinated a total of 4 times, on weeks 0, 2, 4, and 6, while 100 μL of saline was injected into the non-immune saline group rats.

On week 8, rats were anesthetized with Nembutal (100 ml/kg) and intubated for mechanical ventilation. We performed a left thoracotomy to expose the heart and ligated the left anterior descending artery with 6–0 Prolene suture to create an MI; echocardiography was carried out immediately after surgery to verify MI creation. We selected cases based on a 15% or greater reduction in EF compared to preoperative EF.

One week later, we harvested the heart and other organs to examine the effects of the vaccine treatment.

### Hemodynamic measurements

A tail-cuff system (BP-98A, Softron Co., Tokyo, Japan) was used to measure blood pressure and heart at 11 weeks of age in rats without MI induction to evaluate the safety of the sEH vaccine. The rats were allowed to adapt to the apparatus for more than one week before the study.

### Echocardiography

Transthoracic echocardiography was performed in the rats before and after surgery on day 0 and day 7 using an echocardiography machine with a 15-MHz transducer (Toshiba, Japan). M-mode was used for left ventricular echocardiographic recording. Using 2-dimensional short-axis imaging with the M-mode as described previously^34^, we measured the thickness of the anterior wall, diastolic dimension of the left ventricle (LVDd), and LVDs. FS (%) was calculated as (LVDd-LVDs)/LVDd × 100%. The left ventricular EF (%) was calculated as [(LVDd)^3^ − (LVDs)^3^/(LVDd)^3^ × 100%]. Similar and different parts of the heart were measured twice, and averaged data were obtained.

### Histopathological examination

Hearts were harvested from the rats after the echocardiogram examination at one week after MI (day 7). The hearts were fixed in formalin, and sections 2 mm below the LAD ligation level were Azan-stained to make fibrotic areas appear blue (Fig. 3). The ratio of blue-stained area to overall heart muscle area^34,42^ was calculated with ImageJ^43^ and taken as percent fibrosis. The investigators were blinded to treatment.

Antigen retrieval was performed in Dako pH 9 EDTA buffer (Dako, Kyoto, Japan) with a microwave oven and a pressure cooker. Blood vessels were observed by staining for vascular endothelial cells and smooth muscle, with CD31 (abcam, ab28364, 1:100 dilution) and α-smooth muscle actin (α-SMA) (A2547, 1: 1000 dilution) (Sigma Chemical Co. Lt. Louis, MO), respectively. *Griffonia simplicifolia* Lectin I- Isolectin B4 (Lectin) (FL-1201, 1:3000 dilution; Vector Laboratories Ltd., Peterborough, UK) was used to visualize endothelial cells. Hoechst 33342 dye (Sigma, St Louis, MO) was used to stain nuclei. We analyzed these data with HALO AI (Indica Labs, NM).

In addition, we evaluated the safety of the sEH vaccine in rats without MI induction with histopathology. The lung, liver, and kidney were harvested from both control and vaccinated rats at 13 weeks of age. We stained them with hematoxylin and eosin after formalin fixation to determine whether there were adverse pathological changes in the vaccinated group^28^.

### Western blotting

Heart sections were homogenized in RIPA buffer with protease inhibitors. Samples of heart lysates were resolved on SDS-PAGE according to a standard protocol. After transferring protein to membranes, the membranes were probed with primary antibodies followed by secondary antibodies conjugated to horseradish peroxidase, and immunoreactive bands were developed with ECL Plus Western Blotting Detection Reagents (GE Healthcare, UK). Enhanced chemiluminescence was detected with FUSION FX (Vilber-Lourmat, France), and band density was quantified with Image J software. We used the following primary antibodies: β-actin I-19 (sc-1616, 1:1000) and VEGF (A-20) (sc-152, 1: 200) from Santa Cruz Biotechnology (Santa Cruz, CA), and eNOS (#9586, 1: 200) from Cell Signaling Technology (Danvers, MA).

### Statistical analyses

All data are expressed as mean ± SEM. Statistical analyses were conducted by one-way analysis of variance followed by Tukey's test or a two-tailed Student's *t*-test. All statistical analyses were performed using Excel and JMP software programs (SAS Institute, Cary, NC).

## Supplementary Information

Supplementary Information.
