# Author Correction: A novel soluble epoxide hydrolase vaccine protects murine cardiac muscle against myocardial infarction

**DOI:** 10.1038/s41598-022-12232-2

**Published:** 2022-05-11

**Authors:** Takahiro Kitsuka, Aya Shiraki, Jun‑ichi Oyama, Hironori Nakagami, Atsushi Tanaka, Koichi Node

**Affiliations:** 1 Department of Cardiovascular Medicine, Saga University, 5‑1‑1 Nabeshima, Saga, 849‑8501 Japan; 2 Department of Health Development and Medicine, Osaka University Graduate School of Medicine, Osaka, Japan

Correction to: *Scientific Reports* https://doi.org/10.1038/s41598-022-10641-x, Published on 28 April 2022

The original version of this Article contained errors in Figure 6 where the labels for the y-axis were incorrectly given in Japanese characters in Panels (b) and (c). The original Figure 6 and accompanying legend appear below.

The original Article has been corrected.

**Figure 6:**
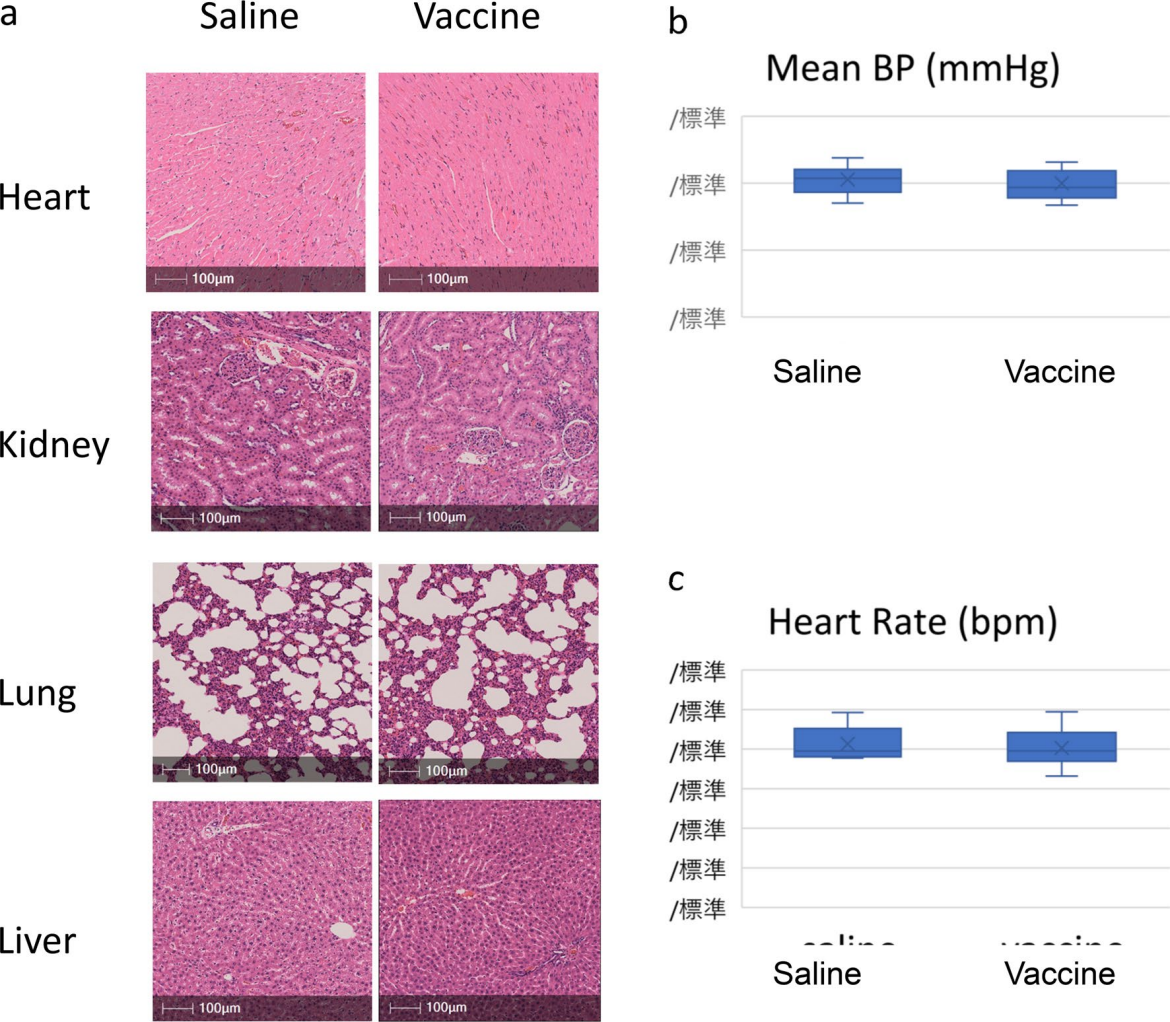
sEH vaccine was safely administrated in rats. (**a**) A representative photomicrograph of hematoxylin and eosin staining in heart, lung, kidney, and liver. Scale bars = 100 μm. (**b**) The mean blood pressure at 11 weeks of age before MI induction. Saline group (n = 10), vaccine group (n = 11). (**c**) The mean heart rates at 11 weeks of age before MI induction. Saline group (n = 10), vaccine group (n = 11).

