# Supplementary material for: A novel soluble epoxide hydrolase vaccine protects murine cardiac muscle against myocardial infarction
Source: Sci Rep. 2022 Apr 28;12:6923. doi: 10.1038/s41598-022-10641-x (PMC9051153; doi:10.1038/s41598-022-10641-x)
Supplement: Supplementary file 1 — Supplementary Information. [file 41598_2022_10641_MOESM1_ESM.pptx]

## Slide 1
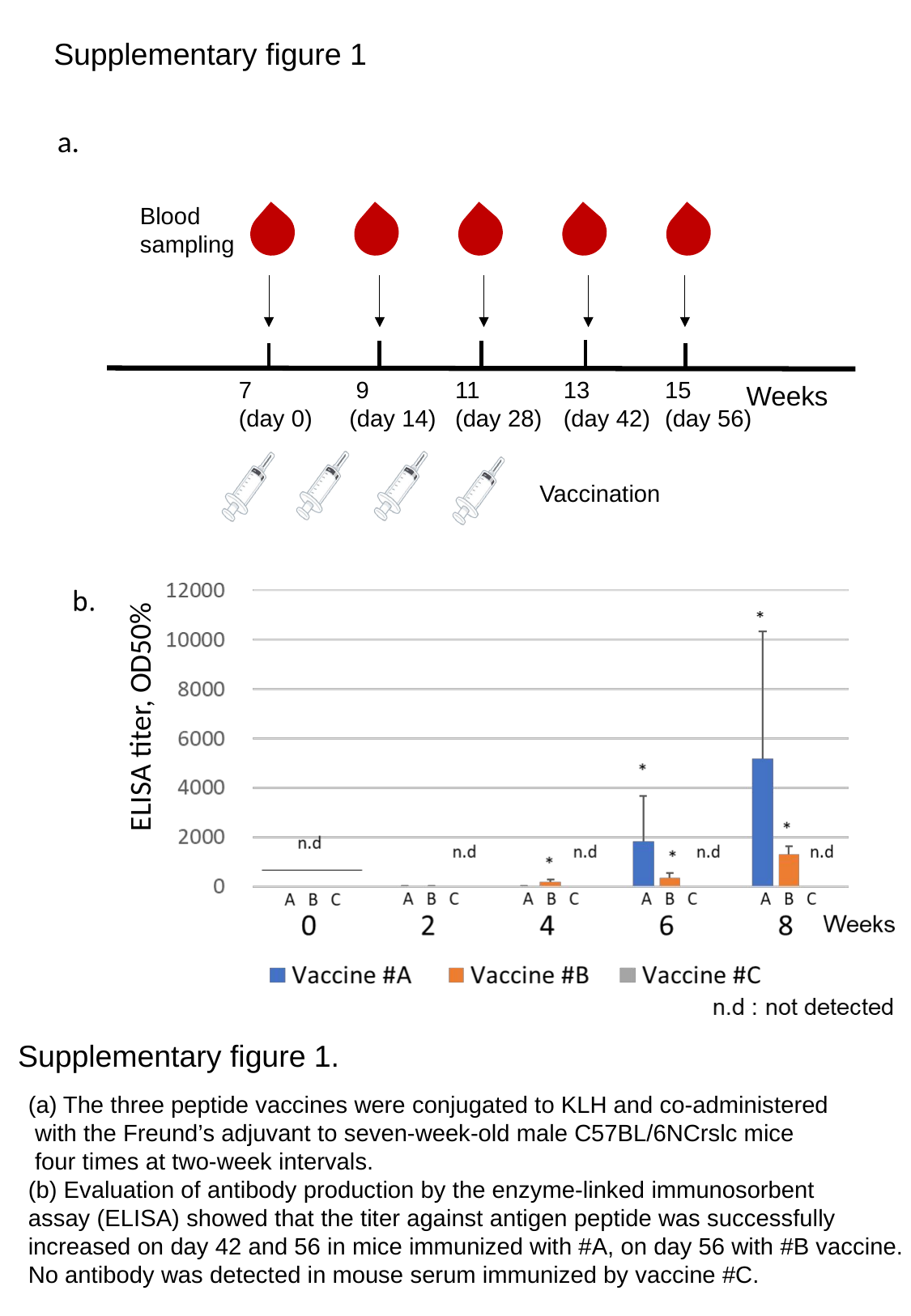

Supplementary figure 1
a.
Blood
sampling
7
(day 0)
 9
(day 14)
11
(day 28)
13
(day 42)
15
(day 56)
Weeks
Vaccination
ELISA titer, OD50%
Supplementary figure 1.
(a) The three peptide vaccines were conjugated to KLH and co-administered
 with the Freund’s adjuvant to seven-week-old male C57BL/6NCrslc mice
 four times at two-week intervals.
(b) Evaluation of antibody production by the enzyme-linked immunosorbent
assay (ELISA) showed that the titer against antigen peptide was successfully
increased on day 42 and 56 in mice immunized with #A, on day 56 with #B vaccine.
No antibody was detected in mouse serum immunized by vaccine #C.

## Slide 2
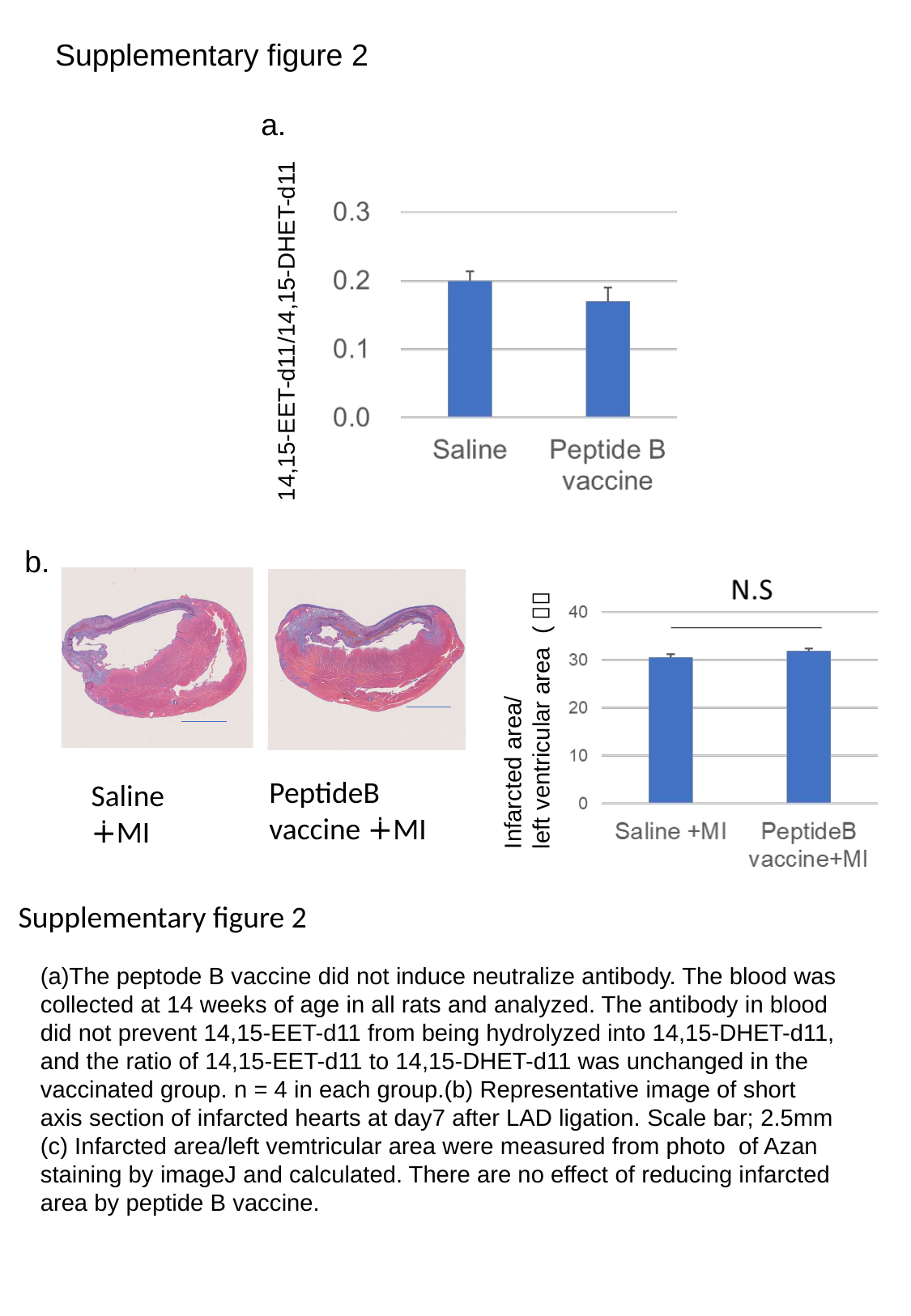

Supplementary figure 2
a.
14,15-EET-d11/14,15-DHET-d11
Infarcted area/
left ventricular area (％）
b.
c
PeptideB vaccine ∔MI
Saline ∔MI
Supplementary figure 2
(a)The peptode B vaccine did not induce neutralize antibody. The blood was collected at 14 weeks of age in all rats and analyzed. The antibody in blood did not prevent 14,15-EET-d11 from being hydrolyzed into 14,15-DHET-d11, and the ratio of 14,15-EET-d11 to 14,15-DHET-d11 was unchanged in the vaccinated group. n = 4 in each group.(b) Representative image of short axis section of infarcted hearts at day7 after LAD ligation. Scale bar; 2.5mm
(c) Infarcted area/left vemtricular area were measured from photo of Azan staining by imageJ and calculated. There are no effect of reducing infarcted area by peptide B vaccine.

## Slide 3
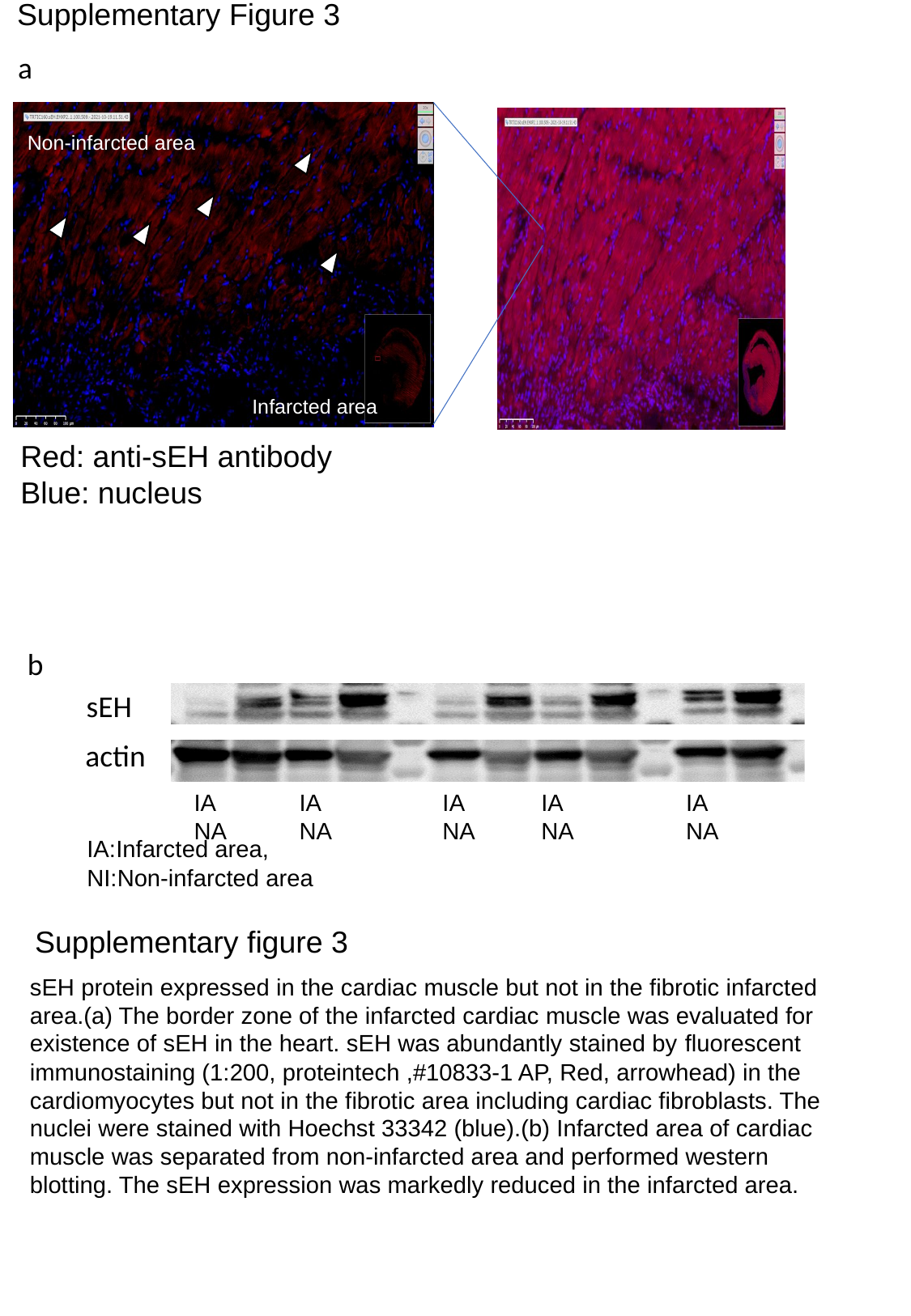

Supplementary Figure 3
a
Non-infarcted area
Infarcted area
Red: anti-sEH antibody
Blue: nucleus
b
sEH
actin
IA　NA
IA　NA
IA　NA
IA　NA
IA　NA
IA:Infarcted area, NI:Non-infarcted area
Supplementary figure 3
sEH protein expressed in the cardiac muscle but not in the fibrotic infarcted area.(a) The border zone of the infarcted cardiac muscle was evaluated for existence of sEH in the heart. sEH was abundantly stained by fluorescent immunostaining (1:200, proteintech ,#10833-1 AP, Red, arrowhead) in the cardiomyocytes but not in the fibrotic area including cardiac fibroblasts. The nuclei were stained with Hoechst 33342 (blue).(b) Infarcted area of cardiac muscle was separated from non-infarcted area and performed western blotting. The sEH expression was markedly reduced in the infarcted area.

## Slide 4
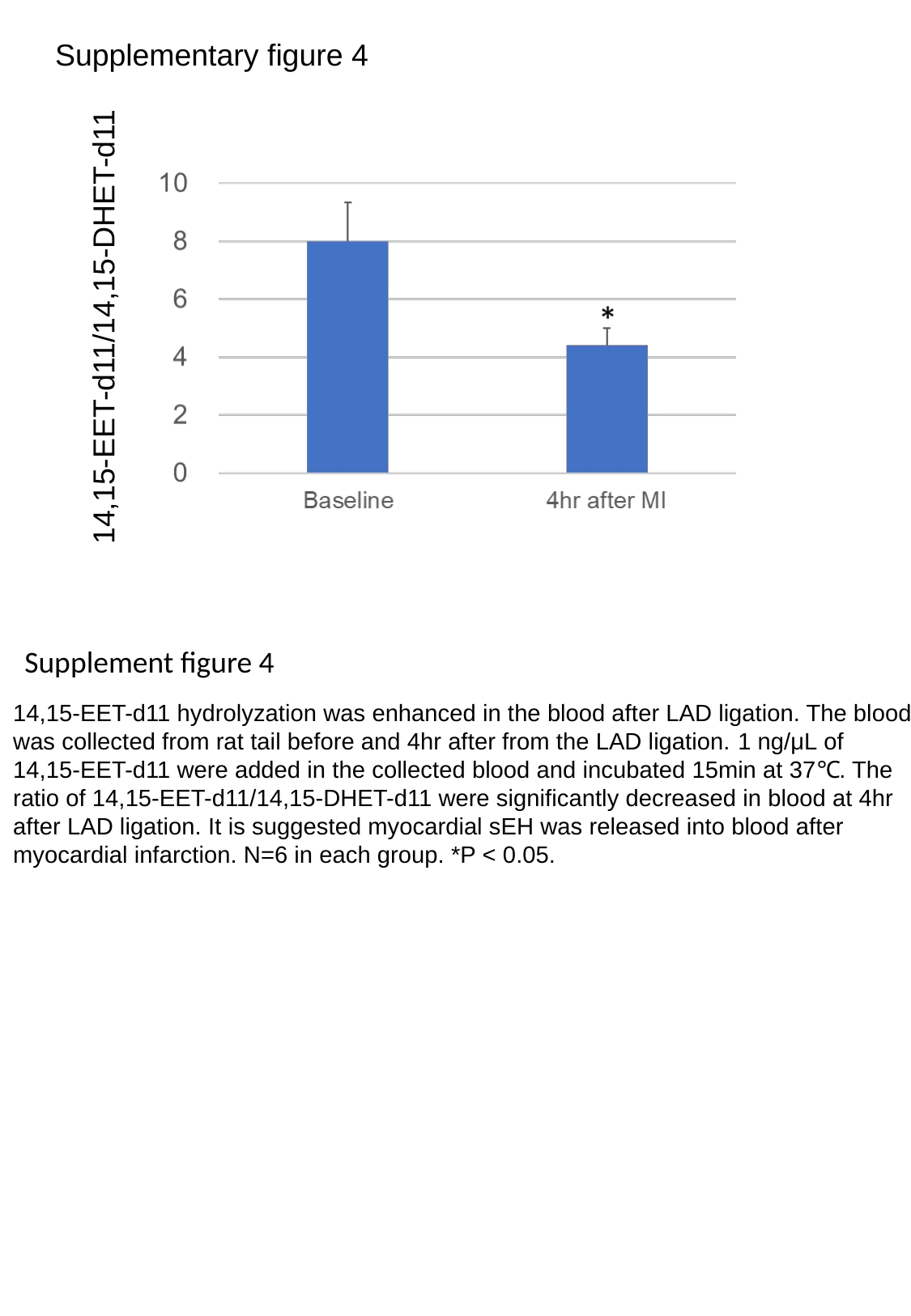

Supplementary figure 4
14,15-EET-d11/14,15-DHET-d11
Supplement figure 4
14,15-EET-d11 hydrolyzation was enhanced in the blood after LAD ligation. The blood
was collected from rat tail before and 4hr after from the LAD ligation. 1 ng/μL of
14,15-EET-d11 were added in the collected blood and incubated 15min at 37℃. The
ratio of 14,15-EET-d11/14,15-DHET-d11 were significantly decreased in blood at 4hr
after LAD ligation. It is suggested myocardial sEH was released into blood after
myocardial infarction. N=6 in each group. *P < 0.05.

## Slide 5
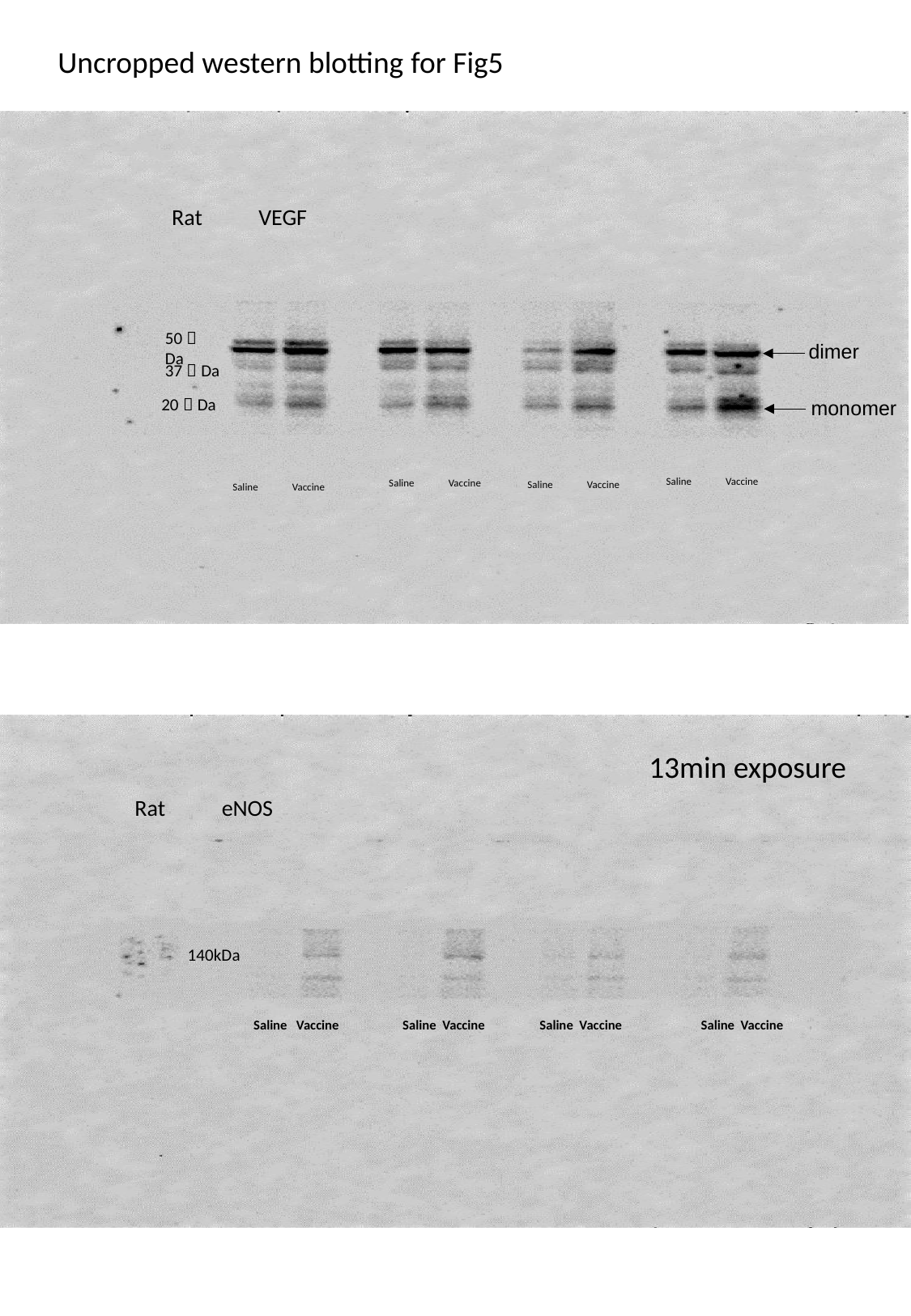

Uncropped western blotting for Fig5
Rat　　VEGF
50ｋDa
37ｋDa
20ｋDa
Saline Vaccine
dimer
monomer
Saline Vaccine
Saline Vaccine
Saline Vaccine
13min exposure
Rat　　eNOS
140kDa
Saline Vaccine Saline Vaccine Saline Vaccine Saline Vaccine

## Slide 6
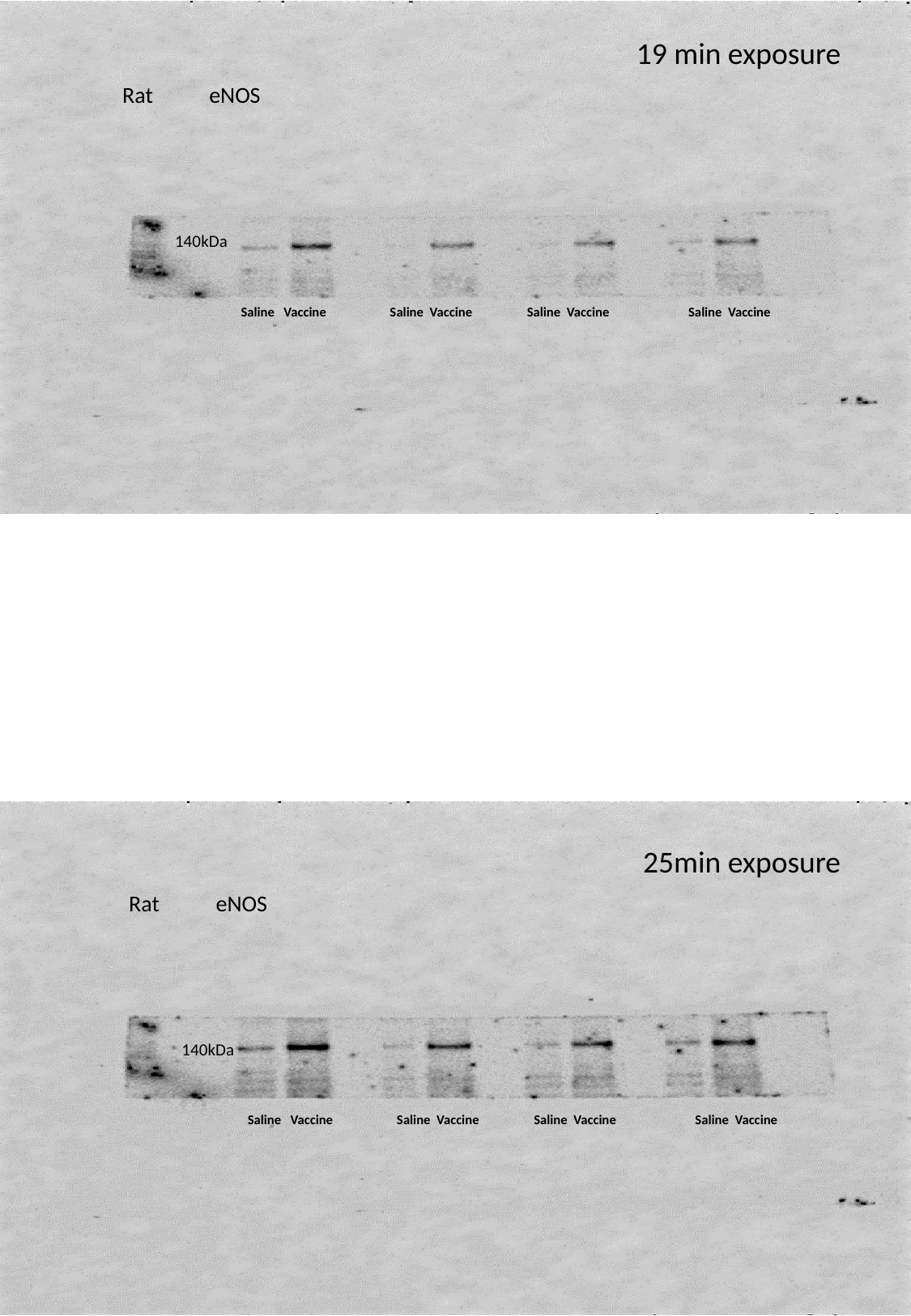

19 min exposure
Rat　　eNOS
140kDa
Saline Vaccine Saline Vaccine Saline Vaccine Saline Vaccine
25min exposure
Rat　　eNOS
140kDa
Saline Vaccine Saline Vaccine Saline Vaccine Saline Vaccine

## Slide 7
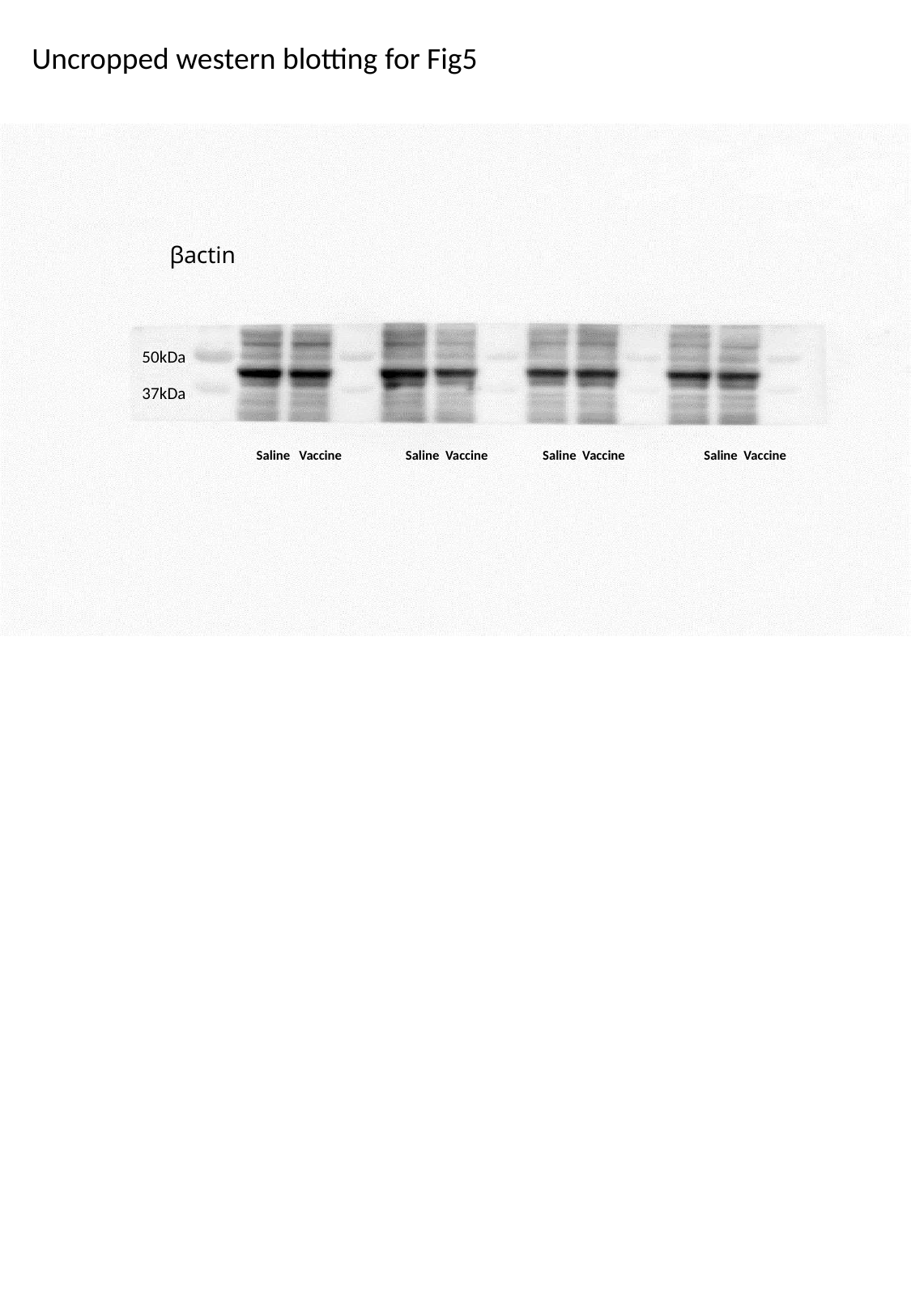

Uncropped western blotting for Fig5
βactin
50kDa
37kDa
Saline Vaccine Saline Vaccine Saline Vaccine Saline Vaccine

## Slide 8
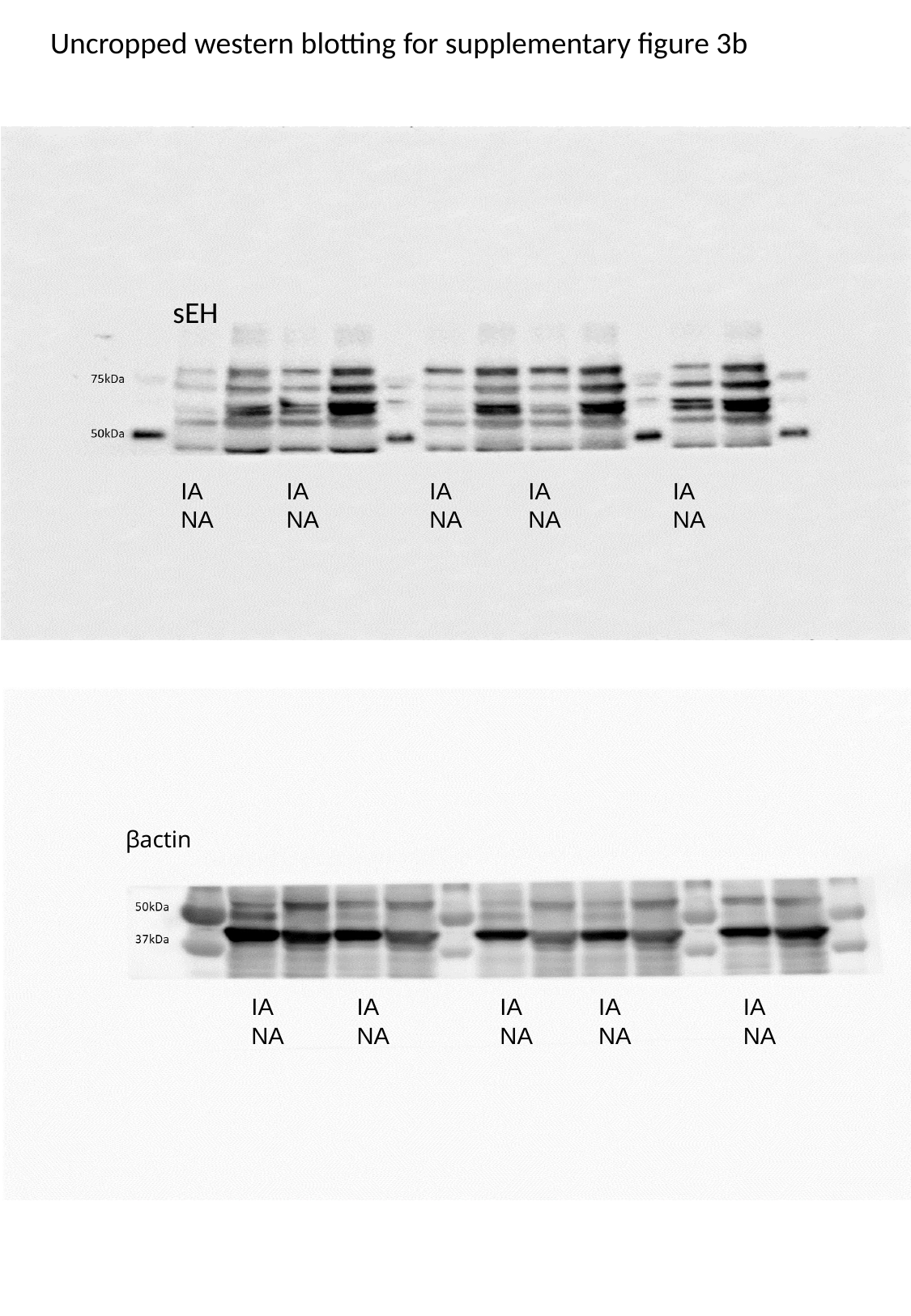

Uncropped western blotting for supplementary figure 3b
sEH
IA　NA
IA　NA
IA　NA
IA　NA
IA　NA
βactin
IA　NA
IA　NA
IA　NA
IA　NA
IA　NA
